# Supplementary material for: All-Ceramic Passive Wireless Temperature Sensor Realized by Tin-Doped Indium Oxide (ITO) Electrodes for Harsh Environment Applications
Source: Sensors (Basel). 2022 Mar 10;22(6):2165. doi: 10.3390/s22062165 (PMC8950959; doi:10.3390/s22062165)
Supplement: Supplementary file 1 [file sensors-22-02165-s001.zip › sensors-1604708-supplementary.pdf]

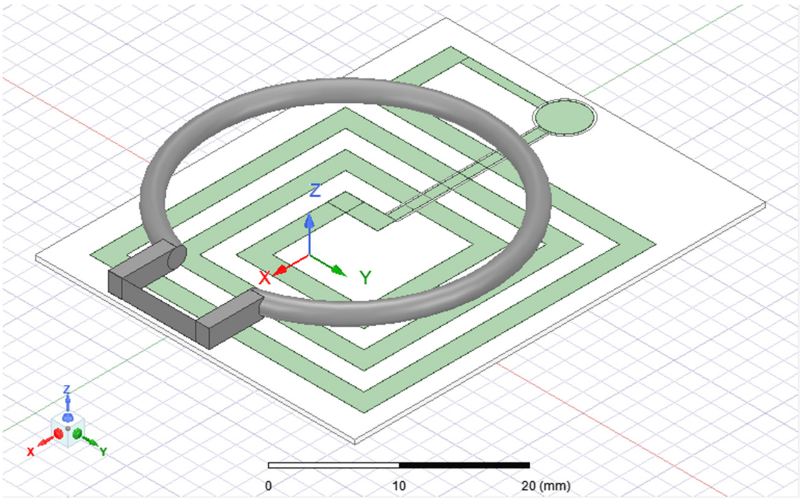

Figure S1. Schematic of the LC resonator model in ANSYS HFSS for finite element analysis. The metallic ring was given Pt electric boundary conditions to mimic the interrogator antenna used in the experimental analysis. The reflection loss (S_{11}) parameter at the Pt antenna was simulated to characterize the resonant frequency of the LC resonator model.
